# Correlation and agreement between the body mass index and abdominal perimeter with the waist-to-height ratio in peruvian adults aged 18 to 59 years

**DOI:** 10.17843/rpmesp.2022.394.11932

**Published:** 2022-12-15

**Authors:** Juan Pablo Aparco, Haydeé Cárdenas-Quintana

**Affiliations:** 1 Doctoral Program in Nutrition, Postgraduate School, Universidad Nacional Agraria La Molina, Lima, Peru. Universidad Nacional Agraria La Molina Doctoral Program in Nutrition Postgraduate School Universidad Nacional Agraria La Molina Lima Peru; 2 National Food and Nutrition Center, Instituto Nacional de Salud, Lima, Peru. National Food and Nutrition Center Instituto Nacional de Salud Lima Peru; 3 Nutrition Department, Universidad Nacional Agraria La Molina, Lima, Peru. Universidad Nacional Agraria La Molina Nutrition Department Universidad Nacional Agraria La Molina Lima Peru

**Keywords:** BMI, Obesity, Abdominal Perimeter, Prevalence, Peru, Adults

## Abstract

**Objective.:**

To determine the correlation and diagnostic agreement of body mass index (BMI) and abdominal perimeter (AP) with the waist-to-height ratio (WHtR).

**Materials and methods.:**

A descriptive, cross-sectional, secondary data study was conducted using the anthropometric databases of the Food and Nutrition Surveillance Survey by Adult Life Stages from 18 to 59 years old, 2017-2018, which included 1084 individuals for the geographic domains of Metropolitan Lima, other urban areas, and rural regions. The prevalence of obesity was estimated according to BMI, AP and WHtR. Lin’s correlation coefficient and Cohen’s Kappa were used to determine the correlation and agreement between the three anthropometric measurements.

**Results.:**

According to the BMI, AP, and WHtR criteria, the prevalence of obesity was 26.8%, 50.4% and 85.4%, respectively; the prevalence was higher in women and in those over 30 years of age. The correlation between BMI and AP, as well as between BMI and WHtR was poor; it was moderate between AP and WHtR, with differences between men and women. Furthermore, the agreement between BMI and AP was acceptable, whereas the agreement between BMI vs. WHtR was mild.

**Conclusions.:**

The results regarding correlation and agreement are limited and suggest that they are not interchangeable measures, so it is necessary to evaluate the adequacy of using BMI alone for the diagnosis of obesity in Peru. The limited correlation and agreement was reflected in the different proportions of obesity that range from 26.8% to 85.4% when applying the three criteria.

## INTRODUCTION

Obesity is a highly prevalent global epidemic that generates multiple health problems ^1^. In 2016, the World Health Organization (WHO) reported more than 650 million obese adults. This figure indicates that global obesity almost tripled since 1975 ^2^. In Peru, the obesity trend, according to BMI, in those over 15 years of age is also growing, increasing from 18.3% in 2013 to 25.8% in 2021 ^3^^,^^4^; in addition, chronic noncommunicable diseases were the causes of 70% of the deaths that occurred in 2018 ^5^.

The harmful effects of obesity have been widely described and include several types of conditions, including hormonal, dietary, metabolic, orthopedic, and psychological effects ^6^^,^^7^, which increase the risk of cardiometabolic diseases ^8^. Moreover, in the current pandemic context, obese persons diagnosed with COVID-19 are six times more likely to die compared to persons of normal weight ^9^.

The body mass index (BMI) is the most used criterion for the diagnosis of obesity, despite the fact that it has limitations to define the distribution of body fat and that it must be adjusted for the population with short stature ^10^. Although the WHO recognizes BMI as the most practical method to determine excess weight ^11^^,^^12^, there are other helpful anthropometric measurements that allow overcoming the limitations of BMI, such as the abdominal perimeter (AP) and the waist-to-height ratio (WHtR), since they consider not only the amount of adipose tissue, but also its location ^13^. Excessive accumulation of fatty tissue in the central region is a more important determinant of risk than excess weight itself ^14^^,^^15^.

Some studies in Peru ^16^^,^^17^, using other indicators such as AP, have reported higher prevalence rates than what was estimated with BMI. In addition, a recent study on Peruvian population reported that the WHtR is the best predictor of arterial hypertension and diabetes mellitus compared to BMI and AP ^18^; however, currently, the agreement between these three anthropometric indicators has not been studied. It is important to consider that Peru is one of the countries whose inhabitants have shorter stature ^19^. Using only the BMI for diagnosing obesity could underestimate its prevalence; therefore, the aim of this study was to determine the diagnostic correlation and agreement between BMI, AP, and WHtR, as well as to compare the prevalence of obesity by applying three diagnostic criteria in Peruvian men and women aged 18 to 59 years.

**KEY MESSAGES**

Motivation for the study. The body mass index (BMI) is the most widely used criterion for diagnosing obesity, despite its limitations and the fact that it is not the most accurate for identifying the risks of metabolic diseases. In Peru, the correlation of various anthropometric measures has not been evaluated in a representative sample of adults.

Main findings. The correlation was poor between BMI and abdominal perimeter (AP) and BMI and waist-to-height ratio (WHtR), and moderate between AP and WHtR. In addition, the diagnostic agreement between BMI and AP was acceptable but between BMI and WHtR was mild.

Implications. The results show that the anthropometric measures evaluated are not interchangeable and that the use of BMI should be re-evaluated since there are other indexes that identify the risks of chronic diseases earlier.

## MATERIALS AND METHODS

### Type and design of the study

We conducted a cross-sectional study was conducted that analyzed a secondary database from the Encuesta Vigilancia Alimentaria y Nutricional por Etapa de Vida Adulto de 18 a 59 años 2017-2018 (VIANEV Adultos 2017-2018). The studied population were Peruvian adults of both sexes aged 18 to 59 years. 

The sample of the primary study followed a multistage, probabilistic, and independent design. The Technical Report of the Food and Nutrition Surveillance by Life Stages; Adults 2017-2018 ^20^ refers that the sample size was 1211 adults and that it was calculated independently for each study stratum (Metropolitan Lima, urban and rural rest), by applying the proportions formula, considering an expected proportion of overweight of 37%, a non-response rate of 13% and a confidence level of 95%. The final sample included 1086 adults nationwide. The sample selection was carried out in two stages, specific details of the sample design are available in the Technical Report of the Food and Nutrition Surveillance by Life Stages; Adults 2017-2018 ^20^.

The inclusion criteria for the VIANEV Adults 2017-2018 were: a) adults between 18 to 59 years old registered in the home identification list and b) adults fasting no less than nine hours and no more than 12 hours for biochemical analysis. Likewise, the exclusion criteria were: a) pregnant or puerperal women, b) adults receiving any medication that could affect glucose or lipid profile, c) adults who consumed food before the biochemical evaluation, d) adults with gastrointestinal diseases that could modify their diet, e) adults with anatomical conditions that do not allow correct application of the anthropometric technique (Down syndrome, scoliosis). For the secondary study, persons without weight, height or abdominal perimeter records were excluded from the database.

### Procedures

Data from the Food and Nutrition Surveillance conducted by the National Center for Food and Nutrition (CENAN) were used. Data on weight, height, abdominal perimeter, and age, among others, were taken by standardized anthropometrists using equipment and instruments calibrated by CENAN ^21^; more information on the process can be found in the aforementioned report ^20^.

Authorization from CENAN was requested in order to access the database. However, at the date of publication of this article, this database is already available at: https://datos.ins.gob.pe/dataset/estado-nutricional-en-adultos-de-18-a-59-anos-peru-2017-2018. After obtaining the database, we carried out consistency procedures to verify that the variables of interest had complete data. The analyses were performed with 1047 records after eliminating those that did not have AP data. Then, the variables BMI and WHtR were calculated and categorized according to the criteria described in the variables section of this article. Statistical analysis was performed on the processed database.

### Variables

The body mass index was calculated with the formula created by Quetelet [BMI=weight (kg) ∕ height (m)^2^]. We used the WHO recommendations for adult population ^12^ to obtain the categories of “individuals with obesity and without obesity”: a) BMI < 30: without obesity and b) BMI ≥ 30: with obesity. 

The abdominal perimeter was calculated to diagnose abdominal obesity. Abdominal obesity was considered when the AP was ≥ 94 cm in men and ≥ 88 cm in women ^21^. 

The waist-to-height ratio relates the AP to the height of the individual through a quotient and is defined as central obesity when the ratio is ≥ 0.5 in both males and females ^22^.

### Statistical analysis

The data analysis was performed in the statistical program STATA version 15 considering the complex sample design of the VIANEV 2017-2018 survey. For this we used the svy command, which considers the clusters, stratification, and expansion factor of the referred survey, for prevalence estimates and comparison of anthropometric characteristics between men and women. 

Descriptive statistics are presented as means and standard deviation; a normal distribution was assumed for quantitative variables due to the sample size exceeding 300 observations. In addition, categorical variables are presented with percentages and 95% confidence intervals (95% CI). Differences in age, weight, height, and anthropometric indicators according to sex were evaluated through the mean comparison test, using Wald adjustment for complex samples. In addition, the prevalence of obesity according to sex, age, and geographic domain were compared with the chi-square test, and the prevalence of obesity applying BMI, AP, and WHtR were compared through the z-test for proportions, considering the survey design.

In order to assess the agreement between the diagnoses of obesity by the BMI, AP and WHtR indicators, we applied the Kappa agreement index ^23^ stratified according to sex, and considering the cut-off points proposed by Landis and Koch: < 0: poor, 0 - 0.20: mild, 0.21 - 0.40 low, 0.41 - 0.60: moderate, 0.61 - 0.80: good and 0.81 - 1.0: excellent. In addition, to evaluate the correlation and agreement of the quantitative data, the measurements of BMI, AP and WHtR were standardized in order to determine the absolute agreement between the anthropometric indicators using Lin’s correlation coefficient considering the following criteria: < 0.90: poor, 0.90 - 0.95: moderate, 0.95 - 0.99: substantial and > 0.99 almost perfect ^24^; these measurements were also compared using Spearman’s correlation coefficient between the difference and the mean of the values, evidencing this behavior by means of Bland-Altman graphs. These analyses did not consider the sample design of the survey and were carried out in a stratified manner according to sex, with a significance level of 0.05.

### Ethical aspects

Data collection for the 2017-2018 VIANEV was conducted within the framework of public health surveillance. The study databases were requested from CENAN; the General Directorate provided the authorization for their use. The database is available at: https://datos.ins.gob.pe/dataset/estado-nutricional-en-adultos-de-18-a-59-anos-peru-2017-2018.

## RESULTS

A total of 1,047 people participated in the study, 57.6% of whom were women; the distribution of groups according to age range fluctuated from 21.4% to 28.5%; Metropolitan Lima was the most frequent geographical domain with almost 50% of the participants, followed by Rural regions (Table 1).

**Table 1:** Demographic characteristics of adults aged 18 to 59 years. Peru, 2017-2018

Characteristics	%	95% CI
Sex		
Women	57.6	54.3 - 60.8
Men	42.4	39.2 - 45.6
Age		
18 - 29 years	28.5	25.4 - 31.9
30 - 39 years	26.5	23.4 - 29.7
40 - 49 years	23.6	20.8 - 26.5
50 - 59 years	21.4	18.6 - 24.4
Geographic domain		
Lima and Callao	49.3	45.9 - 52.6
Rural	30.4	27.1 - 33.8
Other urban areas	20.3	18.3 - 22.4

The expansion factor and sample specifications of the 2017-2018 VIANEV survey were included.

Table 2 shows the anthropometric characteristics of the study population as well as comparisons by sex. Significant differences were found in the mean weight and height, with men having a higher figure in than women (71.8 vs. 64.8 kg and 165.2 vs. 152.2 cm, respectively); despite this, the mean BMI and WHtR in women were significantly higher than in men (27.9 vs. 26.2 and 0.60 vs. 0.55, respectively).

**Table 2:** Anthropometric characteristics of the total population and according to sex. Peru, 2017-2018.

Characteristics	Total (n = 1047) Mean (SD)	Men (n = 465) Mean (SD)	Women (n = 582) Mean (SD)	p-value ^a^
Age (years)	38.5 (11.7)	38.3 (12.1)	38.6 (11.3)	0.712
Weight (kg)	67.9 (13.8)	71.8 (13.7)	64.8 (13.0)	< 0.001
Height (cm)	157.9 (0.88)	165.2 (0.67)	152.2 (0.60)	< 0.001
Body mass index (kg/m^2^)	26.5 (4.9)	26.2 (4.4)	27.9 (5.2)	< 0.001
Abdominal perimeter (cm)	90.5 (12.0)	91.6 (12.0)	91.2 (12.1)	0.579
Waist-to-height ratio	0.58 (0.08)	0.55 (0.07)	0.60 (0.08)	< 0.001

a Comparison of means test with Wald adjustment for complex samples, according to sex.

When comparing the prevalence of obesity according to BMI, AP, and WHtR, by sex, age range and geographic domain, the results show that higher proportions of obesity were found in women, the difference being significant for all cases (Table 3); likewise, the highest prevalence of obesity were observed in the older age groups, which was significant for the three anthropometric indicators. The total prevalence of obesity according to BMI, AP and WHtR was 26.8, 50.4 and 85.4%, respectively; these proportions were significantly different among the three anthropometric measures (p < 0.05, Z-test for proportions).

**Table 3:** Prevalence of obesity by three anthropometric criteria according to sex, age and geographic domain. Peru, 2017-2018.

Characteristic	Body mass index (n=1047) % (95% CI)	Abdominal perimeter (n=1047) % (95% CI)	Waist-to-height ratio (n=1047) % (95% CI)
Sex			
Women	30.9 (26.7 - 35.6)	57.4 (52.7 - 61.8)	89.7 (86.6 - 92.1)
Men	18.2 (14.3 - 22.9)	41.4 (36.0 - 47.0)	79.9 (75.4 - 83.8)
p-value ^a^	< 0.001	< 0.001	< 0.001
Age			
18 - 29 years	10.9 (7.4 - 15.7)	24.3 (18.8 - 30.9)	66.1 (59.1 - 72.6)
30 - 39 years	27.0 (21.0 - 33.8)	53.1 (46.1 - 59.9)	89.8 (85.2 - 93.1)
40 - 49 years	36.3 (29.5 - 43.8)	60.9 (53.7 - 67.7)	92.1 (87.2 - 95.2)
50 - 59 years	31.5 (24.7 - 39.1)	68.4 (60.6 - 75.1)	97.3 (93.4 - 98.9)
p-value ^b^	< 0.001	< 0.001	< 0.001
Geographic domain			
Lima and Callao	26.3 (24.2 - 33.1)	50.6 (45.3 - 55.9)	85.6 (81.7 - 88.7)
Other urban areas	28.8 (23.8 - 34.4)	51.6 (45.9 - 57.3)	86.0 (80.2 - 90.3)
Rural	25.8 (20.3 - 32.6)	48.9 (41.6 - 56.3)	84.1 (79.8 - 87.6)
p-value ^a^	0.623	0.837	0.825
Total	26.8 (22.3 - 29.2)	50.4 (46.7 - 54.0)	85.4 (82.8 - 87.8)

The expansion factor and sample specifications of the 2017-2018 VIANEV survey were included.

a Pearson’s chi-square test, comparison by sex and geographic domain.

b Linear trend chi-square test, comparison by age group.

The diagnostic agreement between BMI and AP was found to be acceptable, although according to sex, men showed moderate agreement (0.49), while women only achieved mild agreement (0.16); the diagnostic agreement between BMI and WHtR was mild in all cases (Table 4).

**Table 4:** Agreement between the diagnosis of obesity by body mass index with abdominal perimeter and waist-to-height ratio, according to sex. Peru, 2018.

Indicator	Agreement (%)	Cohen’s Kappa	Standard error	p-value	Agreement level
Abdominal perimeter					
Sex					
Women	47.2	0.162	0.023	< 0.001	Mild
Men	77.8	0.499	0.040	< 0.001	Moderate
Total	60.8	0.312	0.022	< 0.001	Acceptable
Waist-to-height ratio					
Sex					
Women	40.0	0.091	0.017	< 0.001	Mild
Men	40.0	0.117	0.021	< 0.001	Mild
Total	40.0	0.111	0.014	< 0.001	Mild

The correlation between the standardized measurements of BMI and AP was poor; however, regarding sex, a moderate correlation was found in men, although it was poor in women (0.98 and 0.87, respectively). In the case of the BMI and WHtR standardized measurements, correlation was also found to be poor and the same differences were obtained according to sex (0.90 in men and 0.86 in women). As for the correlation between standardized AP and WHtR, we found a moderate correlation; men showed a poor correlation (0.89), and women a moderate correlation (0.92). Regarding the three pairs of comparisons at the general level, the correlation between AP and WHtR was higher than those between BMI vs WHtR and BMI vs AP (Table 5).

**Table 5:** Correlation measures and absolute agreement between standardized measurements of BMI, AP and WHtR, according to sex. Peru, 2018.

	Lin	Rho	p-value
BMIn and APn			
Men	0.908	0.292	<0.001
Women	0.879	-0.036	0.387
General	0.891	0.081	0.009
BMIn and WHtRn			
Men	0.900	0.028	0.546
Women	0.863	0.014	0.741
General	0.882	0.063	0.042
APn and WHtRn			
Men	0.899	-0.312	<0.001
Women	0.929	0.059	0.153
General	0.916	0.021	0.490

BMIn: standardized body mass index values.

APn: standardized values for abdominal perimeter.

WHtRn: standardized values of waist-height index.

Lin: Lin’s concordance correlation coefficient for absolute agreement.

Rho: Spearman’s correlation coefficient between the difference and the mean.

**Figure 1:**
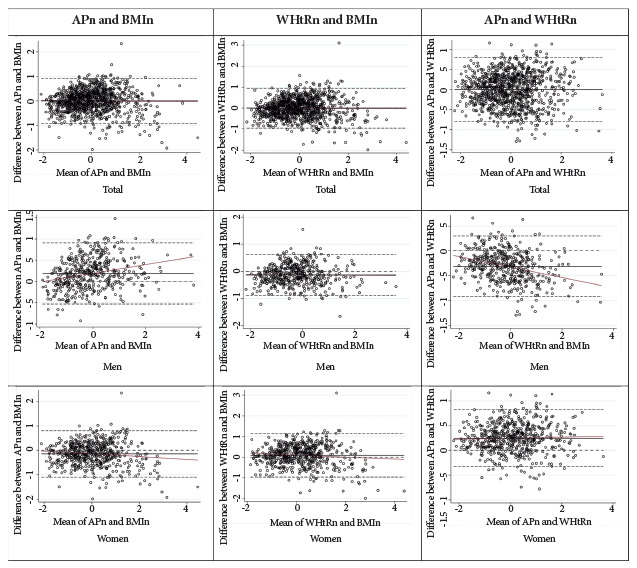
95% Bland-Altman limits of agreement between standardized values of body mass index, abdominal perimeter, and waist-to-height ratio for the total population and according to sex. Peru, 2018.

The Bland-Altman plot in Figure 1 shows no relationship between the mean of the standardized figures of AP and BMI and the difference between the two indicators in the total population; however, for men, the difference increases as the AP and BMI means increase, whereas for women, it is the opposite. As for the standardized values of WHtR and BMI, a relationship was only found in women; thus, the difference between indicators decreases when the means increase. Finally, when analyzing the standardized figures of AP and BMI, we found that in the case of men, the values of BMI increase the higher the measurements of these indicators are, while women show a slightly increasing behavior.

## DISCUSSION

Our results show that the correlations between BMI with AP and WHtR were poor, with differences between men and women according to anthropometric indicator, while the diagnostic agreement between BMI and AP was moderate and that between BMI and WHtR was poor. The prevalence of obesity was significantly different when applying the three diagnostic criteria: according to BMI the prevalence was 26.8%, according to AP it was 50.4%, and when WHtR was used it was 85.4%.

The degree of agreement found by the present study is slightly lower than that reported by Villca *et al*. in Bolivia; they evaluated the agreement between BMI, AP and WHtR and found an agreement index of 0.34 between BMI and AP, and 0.28 between BMI and WHtR ^25^. A systematic review that used Pearson’s coefficient to evaluate correlations and agreement between various anthropometric measures concluded that there was a strong correlation between BMI and AP, as well as between BMI and WHtR; while the agreement was moderate between these anthropometric indicators ^26^. Other studies that have analyzed the relationship between BMI and various anthropometric indicators and even imaging and bioimpedance techniques found similar results. In Belgium, Wilmet *et al*. ^27^ reported a correlation between BMI and AP of 0.91 for men and 0.88 for women (p < 0.05); while in Nigeria, Chinedu *et al*. found a correlation of 0.63 between BMI and AP in the adult population ^28^.

In the present study, BMI showed a mild or insignificant agreement with the WHtR; however, the latter has been recognized as the most accurate index for the early detection of arterial hypertension, diabetes mellitus and metabolic syndrome, as well as other cardiovascular diseases ^29^; this poor agreement of BMI could reflect the limitations of its use for the assessment of body adiposity in the Peruvian population. The applicability of the WHtR cut-off point of 0.5 to diagnose obesity in different ages, sexes and ethnicities has been questioned; in this regard, the systematic review by Browning *et al*., including 78 studies in 14 countries, found that the WHtR score, which encompasses all cardiovascular and metabolic risks, was 0.50 for both sexes ^22^; in addition, height represents the main variation in the anthropometric indicators and differs according to age, sex, and ethnicity. Height is included in the WHtR, so it would reflect this variation and modify the interpretation of the abdominal perimeter adjusted to the height of the individual.

Our results also show moderate correlation between BMI, AP and WHtR and moderate agreement with AP, which would indicate that the estimates with these anthropometric indicators are not interchangeable, even more so when differences are found between men and women. The implications of these results include clinical aspects such as the application of anthropometric indices complementary to BMI for earlier detection of the population at risk of noncommunicable diseases; as well as the need to review and determine the most sensitive BMI cut-off points for predicting cardiometabolic diseases. The BMI is the anthropometric index most commonly used to determine obesity and indirectly the amount of adipose tissue, although it does not discriminate lean tissue from fat ^12^. Recent literature on the diagnosis of obesity recommends the use of abdominal obesity indexes because they assess the location of fat and identify the excess of central fat, providing an opportunity for early diagnosis of metabolic diseases ^30^. Therefore, it is necessary to evaluate the nutritional status considering abdominal obesity indicators to improve the diagnosis, in order to achieve early identification of the population that requires treatment and to rigorously evaluate the interventions and public policies against obesity.

Other studies have estimated the prevalence of obesity by applying the various anthropometric indices and have reported different results. In Australia Booth *et al*. reported higher prevalence rates of obesity when applying AP and WHtR criteria compared to BMI ^31^; Myung *et al*. found prevalence rates of obesity of 3.6%, 26.2% and 43.3% when applying BMI, AP, and WHtR, respectively ^32^. Likewise, a study in Peru reported lower prevalence rates of obesity when using BMI, but the figures were two to three times higher when applying AP and WHtR ^18^. These findings suggest that the proportion of obesity by BMI may be underestimated in Peru and that it is necessary to analyze the specificity of the BMI cut-off point for the Peruvian population, as well as to use complementary indicators to BMI for the diagnoses of obesity.

One of the limitations of the study was that we did not include a gold standard test to establish an accurate diagnosis of obesity. Additionally, we only used three diagnostic techniques, but there are other more precise techniques such as X-ray photon absorptiometry (DEXA); likewise, the cut-off points for AP and WHtR are still under discussion in Peru. Another aspect to consider was that the measurements between the three diagnostic criteria use different units of measurement, which makes a direct comparison difficult, so we decided to standardize the variables of BMI, AP and WHtR.

In conclusion, this study found that BMI correlated moderately with AP and WHtR, with differences according to sex; likewise, the agreement was moderate between BMI and AP but insignificant with WHtR, which is the best predictor of cardiovascular disease and hypertension. Our results suggest that correlation and agreement are not interchangeable indicators and that it is necessary to evaluate the sufficiency of using BMI alone for the diagnosis of obesity in our country. Furthermore, the prevalence rates of obesity in Peru were significantly different when applying each diagnostic criterion. We recommend the use of anthropometric indices that estimate abdominal obesity complementary to BMI.

## References

[1] Organización Mundial de la Salud (2017). Obesidad y sobrepeso. Centro de prensa. Nota descriptiva.

[2] NCD Risk Factor Collaboration (NCD-RisC) (2017) Worldwide trends in body-mass index, underweight, overweight, and obesity from 1975 to 2016: a pooled analysis of 2416 population-based measurement studies in 128·9 million children, adolescents, and adults. Lancet.

[3] Instituto Nacional de Estadística e Informática (2014). Encuesta Nacional de Demografía y Salud. ENDES 2013.

[4] Instituto Nacional de Estadistica e Informática (2021). Encuesta Nacional de Demografía y Salud ENDES 2020.

[5] World Health Organization (WHO) (2019). Noncommunicable diseases country profiles 2018.

[6] Lobstein T, Baur L, Uauy R, IASO International Obesity TaskForce (2004). Obesity in children and young people a crisis in public health. Obes Rev.

[7] World Health Organization (WHO) (2000). Obesity: preventing and managing the global epidemic. Report of a WHO consultation.

[8] Han TS, Sattar N, Lean M (2006). ABC of obesity Assessment of obesity and its clinical implications. BMJ.

[9] Gao F, Zheng KI, Wang XB, Sun QF, Pan KH, Wang TY (2020). Obesity Is a Risk Factor for Greater COVID-19 Severity. Diabetes Care.

[10] Garn SM, Leonard WR, Hawthorne VM (1986). Three limitations of the body mass index. Am J Clin Nutr.

[11] World Health Organization (WHO) (2011). Waist circumference and waist-hip ratio: report of a WHO Expert Consultation Geneva.

[12] World Health Organization (1995). Physical status: the use and interpretation of anthropometry. WHO Technical Report Series 854.

[13] Ho SY, Lam TH, Janus ED, Hong Kong Cardiovascular Risk Factor Prevalence Study Steering Committee (2003). Waist to stature ratio is more strongly associated with cardiovascular risk factors than other simple anthropometric indices. Ann Epidemiol.

[14] Nishida C, Ko GT, Kumanyika S (2010). Body fat distribution and noncommunicable diseases in populations overview of the 2008 WHO Expert Consultation on Waist Circumference and Waist-Hip Ratio. Eur J Clin Nutr.

[15] Barbosa PJ, Lessa I, de Almeida Filho N, Magalhães LB, Araújo J (2006). Criteria for central obesity in a Brazilian population impact on metabolic syndrome. Arq Bras Cardiol.

[16] Pajuelo J, Torres L, Agüero R, Bernui I (2019). Sobrepeso y obesidad en la población adulta del Perú. An Fac med.

[17] Carrillo-Larco RM, Miranda JJ, Gilman RH, Checkley W, Smeeth L, Bernabé-Ortiz A (2018). Trajectories of body mass index and waist circumference in four Peruvian settings at different level of urbanisation the CRONICAS Cohort Study. J Epidemiol Community Health.

[18] Hernández-Vásquez A, Azañedo D, Vargas-Fernández R, Aparco JP, Chaparro RM, Santero M (2020). Cut-off points of anthropometric markers associated with hypertension and diabetes in Peru Demographic and Health Survey 2018. Public Health Nutr.

[19] NCD Risk Factor Collaboration (NCD-RisC) (2016). A century of trends in adult human height. Elife.

[20] INS/CENAN (2021). Informe técnico de la Vigilancia Alimentaria y Nutricional por Etapas de Vida; Adultos 2017-2018.

[21] (2012). Guía técnica para la valoración nutricional antropométrica de la persona adulta.

[22] Browning LM, Hsieh SD, Ashwell M (2010). A systematic review of waist-to-height ratio as a screening tool for the prediction of cardiovascular disease and diabetes 0·5 could be a suitable global boundary value. Nutr Res Rev.

[23] Landis JR, Koch GG (1977). The Measurement of Observer Agreement for Categorical Data. Biometrics.

[24] McBride GB A proposal for strength-of-agreement criteria for Lin's concordance correlation coefficient," NIWA Client.

[25] Villca Villegas JL, Chavez-Soliz HR, Mamani Ortiz Y, Arévalo Gonzales MR (2019). Correlación y concordancia de los índices circunferencia/cintura y circunferencia/talla con el índice de masa corporal. Gac Med Bol.

[26] Mahmoud I, Al-Wandi AS, Gharaibeh SS, Mohamed SA (2021). Concordances and correlations between anthropometric indices of obesity a systematic review. Public Health.

[27] Wilmet G, Verlinde R, Vandevoorde J, Carnol L, Devroey D (2017). Correlation between Body Mass Index and abdominal circumference in Belgian adults a cross-sectional study. Rom J Intern Med.

[28] Chinedu SN, Ogunlana OO, Azuh DE, Iweala EE, Afolabi IS, Uhuegbu CC (2013). Correlation between body mass index and waist circumference in nigerian adults implication as indicators of health status. J Public Health Res.

[29] Ashwell M, Gunn P, Gibson S (2012). Waist-to-height ratio is a better screening tool than waist circumference and BMI for adult cardiometabolic risk factors systematic review and meta-analysis. Obes Rev.

[30] Lo K, Wong M, Khalechelvam P, Tam W (2016). Waist-to-height ratio, body mass index and waist circumference for screening paediatric cardio-metabolic risk factors a meta-analysis. Obes Rev.

[31] Booth ML, Hunter C, Gore CJ, Bauman A, Owen N (2000). The relationship between body mass index and waist circumference implications for estimates of the population prevalence of overweight. Int J Obes Relat Metab Disord.

[32] Myung J, Jung KY, Kim TH, Han E (2019). Assessment of the validity of multiple obesity indices compared with obesity-related co-morbidities. Public Health Nutr.

