# Supplementary material for: Correlation and agreement between the body mass index and abdominal perimeter with the waist-to-height ratio in peruvian adults aged 18 to 59 years
Source: Rev Peru Med Exp Salud Publica. 2022 Dec 15;39(4):392–9. doi: 10.17843/rpmesp.2022.394.11932 (PMC11397726; doi:10.17843/rpmesp.2022.394.11932)
Supplement: Translation

# Translation: Correlación y concordancia del índice de masa corporal con el perímetro abdominal y el índice cintura-talla en adultos peruanos de 18 a 59 años

**Authors:** Juan Pablo Aparco, Haydeé Cárdenas-Quintana

**Affiliations:** 1 Programa Doctoral en Nutrición, Escuela de Posgrado, Universidad Nacional Agraria La Molina, Lima, Perú.; 2 Centro Nacional de Alimentación y Nutrición, Instituto Nacional de Salud, Lima, Perú.; 3 Departamento de Nutrición, Universidad Nacional Agraria La Molina, Lima, Perú.

**Keywords:** IMC, Obesidad, Perímetro Abdominal, Prevalencia, Perú, Adultos

## Abstract

**Objetivos.:**

Determinar la correlación y concordancia diagnóstica del índice de masa corporal (IMC), con el perímetro abdominal (PA) y el índice cintura-talla (ICT).

**Materiales y métodos.:**

Se realizó un estudio descriptivo, transversal, de datos secundarios usando las bases de datos antropométricos de la Encuesta Vigilancia Alimentaria y Nutricional por Etapas de Vida Adulto de 18 a 59 años 2017-2018 que incluyó 1084 personas para los dominios geográficos Lima Metropolitana, resto urbano y rural. Se estimaron las prevalencias de obesidad según IMC, PA e ICT y se empleó el coeficiente de correlación de Lin y Kappa de Cohen para determinar la correlación y concordancia entre las tres mediciones antropométricas.

**Resultados.:**

Según los criterios de IMC, PA e ICT las prevalencias de obesidad fueron de 26,8%, 50,4% y 85,4%, respectivamente; las prevalencias fueron mayores en mujeres y en mayores de 30 años. La correlación entre IMC vs. PA e IMC vs. ICT fue pobre y de PA con ICT fue moderada, con diferencias entre hombres y mujeres. Además, la concordancia entre IMC y PA fue aceptable, mientras que la concordancia entre IMC vs. ICT fue leve.

**Conclusiones:**

. Los resultados de la correlación y concordancia son limitados y sugieren que no son medidas intercambiables, por lo que es necesario evaluar la suficiencia de emplear solo IMC para el diagnóstico de obesidad en el Perú. Esta limitada correlación y concordancia se refleja en las diferentes proporciones de obesidad que oscilan entre 26,8% a 85,4% al aplicar los tres criterios.

## INTRODUCCIÓN

La obesidad es una epidemia mundial con alta prevalencia que genera múltiples daños a la salud ^1^. En el 2016, la Organización Mundial de la Salud (OMS) reportó más de 650 millones de adultos obesos. Esta cifra indica que la obesidad mundial casi se triplicó desde 1975 ^2^. En Perú la tendencia de la obesidad, según IMC, en mayores de 15 años también es creciente, pues aumentó de 18,3% en el 2013 a 25,8% en el 2021 ^3^ ^,^ ^4^; además, las enfermedades crónicas no trasmisibles fueron las causas del 70% de las muertes ocurridas en el 2018 ^5^. 

Por su parte, los efectos deletéreos de la obesidad han sido ampliamente descritos y abarcan varios tipos de alteraciones, entre ellas hormonales, alimentarias, metabólicas, ortopédicas y psicológicas ^6^ ^,^ ^7^, que incrementan el riesgo de padecer enfermedades cardiometabólicas ^8^. Más aún, en el actual contexto de pandemia, las personas obesas con diagnóstico de COVID-19 tienen seis veces más riesgo de fallecer comparadas con personas de peso normal ^9^.

Para el diagnóstico de la obesidad, el criterio más usado es el índice de masa corporal (IMC), a pesar de que tiene limitaciones para definir la distribución de la grasa corporal y que debe ser adecuado para la población de baja estura ^10^. Si bien la OMS reconoce al IMC como el método más práctico para determinar el exceso de peso ^11^ ^,^ ^12^, existen otras medidas antropométricas que permiten superar las limitaciones del IMC como el perímetro abdominal (PA) y el índice cintura-talla (ICT) porque consideran no solo la cantidad de tejido adiposo, sino también su localización ^13^. La acumulación excesiva de tejido graso en la región central es un determinante de riesgo más importante que el propio exceso de peso ^14^ ^,^ ^15^. 

Algunos estudios en Perú ^16^ ^,^ ^17^, usando otros indicadores como PA, han reportado prevalencias superiores a lo estimado según IMC; además, un reciente estudio en población peruana reportó que el ICT es el mejor predictor de hipertensión arterial y diabetes mellitus comparado con el IMC y el PA ^18^; sin embargo, hasta la fecha no se ha estudiado la concordancia entre estos tres indicadores antropométricos. Considerando que Perú es uno de los países cuyos pobladores tienen menor estatura ^19^. Utilizar solo el IMC para el diagnóstico de obesidad podría subestimar su prevalencia; por lo tanto, el objetivo del presente estudio fue determinar la correlación y concordancia diagnóstica entre el IMC, el PA y el ICT, así como comparar las prevalencias de obesidad aplicando tres criterios diagnósticos en hombres y mujeres peruanas de 18 a 59 años.

**MENSAJES CLAVE**

Motivación para realizar el estudio. El índice masa corporal (IMC) es el criterio más usado para diagnosticar obesidad, a pesar de sus limitaciones y de que no es el más preciso para identificar los riesgos de enfermedades metabólicas. En Perú no se ha evaluado la correlación de diversas medidas antropométricas en una muestra representativa de adultos.

Principales hallazgos. La correlación entre el IMC y el perímetro abdominal (PA) e IMC y el índice cintura-talla (ICT) fue pobre y entre PA e ICT fue moderada. Además, la concordancia diagnóstica entre; IMC y PA fue aceptable y entre IMC e ICT fue leve.

Implicancias. Los resultados muestran que las medidas antropométricas evaluadas no serían intercambiables y que al existir otros índices que identifican más precozmente los riesgos a enfermedades crónicas debe evaluarse el uso del IMC.

## MATERIALES Y MÉTODOS

### Tipo y diseño del estudio

Se realizó un estudio transversal, con análisis de una base de datos secundaria proveniente de la Encuesta Vigilancia Alimentaria y Nutricional por Etapa de Vida Adulto de 18 a 59 años 2017-2018 (VIANEV Adultos 2017-2018). La población de estudio fueron adultos peruanos de ambos sexos de 18 a 59 años.

El diseño de la muestra del estudio primario fue estratificado, multietápico, probabilístico e independiente. El Informe técnico de la Vigilancia Alimentaria y Nutricional por Etapas de Vida; Adultos 2017-2018 ^20^ refiere que el tamaño de muestra fue de 1211 adultos y que su cálculo se efectuó de manera independiente para cada estrato de estudio (Lima Metropolitana, resto urbano y rural), aplicando la fórmula de proporciones, considerando una proporción esperada de sobrepeso de 37%, una tasa de no respuesta de 13% y un nivel de confianza de 95%. La muestra final recogida fue de 1086 adultos a nivel nacional. La selección de la muestra fue en dos etapas, los detalles específicos del diseño muestral están disponibles en el Informe Técnico de la Vigilancia Alimentaria y Nutricional por Etapas de Vida; Adultos 2017-2018 ^20^.

Los criterios de selección de la VIANEV Adultos 2017-2018 fueron: criterios de inclusión: a) adultos entre 18 a 59 años registrados en el listado de identificación de la vivienda y b) adultos en ayunas no menor de nueve horas ni mayor de 12 horas para los análisis bioquímicos. Asimismo, los criterios para exclusión fueron: a) mujeres gestantes o puérperas, b) adultos que reciben alguna medicación que podría afectar la glucosa o perfil lipídico, c) adultos que consumieron alimentos antes de la evaluación bioquímica, d) adultos con enfermedades gastrointestinales que podrían modificar su alimentación, e) adultos con condiciones anatómicas que no permitan aplicar correctamente la técnica antropométrica (síndrome de Down, escoliosis). Para el estudio secundario se excluyeron de la base de datos las personas sin registro de peso, talla o perímetro abdominal. 

### Procedimientos

Los datos provienen de la Vigilancia Alimentaria y Nutricional realizada por el Centro Nacional de Alimentación y Nutrición (CENAN). Los datos de peso, estatura, perímetro abdominal y edad, entre otros fueron tomados por antropometristas estandarizados usando equipos e instrumentos calibrados por el CENAN ^21^, más información del proceso en el referido informe ^20^. 

Para acceder a la base de datos se procedió a solicitar la autorización del CENAN. Sin embargo, a la fecha de publicación del artículo, esta base de datos ya se encuentra disponible en: https://datos.ins.gob.pe/dataset/estado-nutricional-en-adultos-de-18-a-59-anos-peru-2017-2018. Luego de obtener la base de datos, se realizaron procedimientos de consistencia para verificar que las variables de interés tengan observaciones completas. Los análisis se realizaron con 1047 registros después de eliminar aquellos que no tuvieron datos de PA. Luego se calcularon las variables IMC e ICT y se categorizaron según los criterios descritos en variables. Con la base de datos procesada se realizó el análisis estadístico. 

### Variables

El índice de masa corporal se calculó con la fórmula creada por Quetelet [IMC=peso (kg) ∕ estatura (m)^2^]. Para obtener las categorías de «individuos con obesidad y sin obesidad» se utilizó las recomendaciones de la OMS para población adulta ^12^: a) IMC < 30: sin obesidad y b) IMC ≥ 30: con obesidad. 

El perímetro abdominal se calculó para diagnosticar la obesidad abdominal. Se consideró obesidad abdominal cuando el PA fue ≥ 94 cm en varones y ≥ 88 cm en mujeres ^21^. 

El índice cintura-talla relaciona el PA con la talla del individuo a través de un cociente y se define como obesidad central cuando la razón es ≥ 0,5, tanto en hombres como en mujeres ^22^. 

### Análisis estadístico

El análisis de datos se realizó en el programa estadístico STATA versión 15, tomando en cuenta el diseño muestral complejo de la encuesta VIANEV 2017-2018, para lo cual se aplicó el comando svy que considera los conglomerados, la estratificación y el factor de expansión de la referida encuesta, para las estimaciones de prevalencias y comparación de características antropométricas entre hombres y mujeres. 

Las estadísticas descriptivas se presentan como medias y desviación estándar; se asumió una distribución de normalidad en las variables cuantitativas por el tamaño de muestra que supera las 300 observaciones. Además, las variables categóricas se muestran con porcentajes e intervalos de confianza al 95% (IC 95%). Las diferencias de edad, peso, talla, e indicadores antropométricos según sexo se evaluaron a través de la prueba de comparación de medias, mediante ajuste de Wald para muestras complejas; además, las prevalencias de obesidad según sexo, edad y dominio geográfico se compararon a través de la prueba de chi cuadrado y las prevalencias de obesidad aplicando IMC, PA e ICT se compararon a través de la prueba z de proporciones, considerando el diseño de la encuesta.

Para valorar la concordancia entre los diagnósticos de obesidad de los indicadores IMC, PA e ICT se aplicó el índice de concordancia Kappa ^23^ estratificado según sexo y considerando los puntos de corte propuestos por Landis y Koch: < 0: pobre, 0 - 0,20: leve, 0,21 - 0,40 baja, 0,41 - 0,60: moderada, 0,61 - 0,80: buena y 0,81 - 1,0: excelente. Además, para evaluar la correlación y concordancia de los datos cuantitativos de IMC, PA e ICT, las mediciones fueron estandarizadas para determinar el acuerdo absoluto entre los indicadores antropométricos mediante el coeficiente de correlación de Lin considerando los siguientes criterios: < 0,90: pobre, 0,90 - 0,95: moderado, 0,95 - 0,99: sustancial y > 0,99 casi perfecto ^24^; también se compararon estas mediciones empleando el coeficiente de correlación de Spearman entre la diferencia y la media de los valores, evidenciando este comportamiento mediante gráficos de Bland-Altman. Estos análisis se realizaron sin considerar el diseño muestral de la encuesta y de forma estratificada según sexo, y considerando un nivel de significancia de 0,05. 

### Aspectos éticos

La recolección de datos de la VIANEV 2017-2018 se realizó en el marco de la vigilancia en salud pública. Las bases de datos del estudio se solicitaron al CENAN y se obtuvo la autorización de la Dirección General para su uso. La base de datos se encuentra disponible en: https://datos.ins.gob.pe/dataset/estado-nutricional-en-adultos-de-18-a-59-anos-peru-2017-2018.

## RESULTADOS

Participaron del estudio, 1047 personas, de ellas el 57,6% fueron mujeres, la distribución de grupos según rango de edad osciló de 21,4 a 28,5%; mientras que, para el dominio geográfico predominó Lima Metropolitana con casi el 50% de las observaciones, seguido por Resto urbano (Tabla 1).

**Tabla 1:** Características demográficas de adultos de 18 a 59 años. Perú, 2017-2018.

Características	%	IC 95%
Sexo		
Mujer	57,6	54,3 - 60,8
Hombre	42,4	39,2 - 45,6
Edad		
18 - 29 años	28,5	25,4 - 31,9
30 - 39 años	26,5	23,4 - 29,7
40 - 49 años	23,6	20,8 - 26,5
50 - 59 años	21,4	18,6 - 24,4
Dominio geográfico		
Lima y Callao	49,3	45,9 - 52,6
Resto urbano	30,4	27,1 - 33,8
Rural	20,3	18,3 - 22,4

Se incluyó el factor de expansión y las especificaciones muestrales de la encuesta VIANEV 2017-2018.

En la tabla 2 se muestran las características antropométricas de la población de estudio y se comparan según sexo. Destacan con diferencias significativas en las medias de peso, y talla, con un valor más alto en hombres que en mujeres (71,8 vs. 64,8 kg y 165,2 vs. 152,2 cm, respectivamente); a pesar de ello en las mujeres los promedios de IMC e ICT fueron significativamente mayores que en los varones (27,9 vs. 26,2 y 0,60 vs. 0,55, respectivamente).

**Tabla 2:** Características antropométricas de la población total y según sexo. Perú, 2017-2018.

Características	Total (n = 1047) Media (DE)	Hombres (n = 465) Media (DE)	Mujeres (n = 582) Media (DE)	Valor de p ^a^
Edad (años)	38,5 (11,7)	38,3 (12,1)	38,6 (11,3)	0,712
Peso (kg)	67,9 (13,8)	71,8 (13,7)	64,8 (13,0)	< 0,001
Talla (cm)	157,9 (0,88)	165,2 (0,67)	152,2 (0,60)	< 0,001
Índice de masa corporal (kg/m^2^)	26,5 (4,9)	26,2 (4,4)	27,9 (5,2)	< 0,001
Perímetro abdominal (cm)	90,5 (12,0)	91,6 (12,0)	91,2 (12,1)	0,579
Índice cintura-talla	0,58 (0,08)	0,55 (0,07)	0,60 (0,08)	< 0,001

a Prueba de comparación de medias con ajuste de Wald para muestras complejas, comparación según sexo.

Al comparar las prevalencias de obesidad según el IMC, PA e ICT, por sexo, rango de edad y dominio geográfico, los resultados muestran que se encontraron mayores proporciones de obesidad en mujeres, siendo la diferencia significativa en todos los casos (Tabla 3); asimismo en los grupos de mayor edad se observaron las más altas prevalencias de obesidad, lo cual resultó significativo para los tres indicadores antropométricos. La prevalencia total de obesidad según IMC, PA e ICT fue de 26,8, 50,4 y 85,4%, respectivamente, estas proporciones fueron significativamente diferentes entre las tres medidas antropométricas (p < 0,05, prueba Z de proporciones).

**Tabla 3:** Prevalencia de obesidad aplicando tres criterios antropométricos según sexo, edad y dominio geográfico. Perú, 2017-2018.

Característica	Índice de masa corporal (n=1047) % (IC 95%)	Perímetro abdominal (n=1047) % (IC 95%)	Índice cintura-talla (n=1047) % (IC 95%)
Sexo			
Mujer	30,9 (26,7 - 35,6)	57,4 (52,7 - 61,8)	89,7 (86,6 - 92,1)
Hombre	18,2 (14,3 - 22,9)	41,4 (36,0 - 47,0)	79,9 (75,4 - 83,8)
Valor de p ^a^	< 0,001	< 0,001	< 0,001
Edad			
18 - 29 años	10,9 (7,4 - 15,7)	24,3 (18,8 - 30,9)	66,1 (59,1 - 72,6)
30 - 39 años	27,0 (21,0 - 33,8)	53,1 (46,1 - 59,9)	89,8 (85,2 - 93,1)
40 - 49 años	36,3 (29,5 - 43,8)	60,9 (53,7 - 67,7)	92,1 (87,2 - 95,2)
50 - 59 años	31,5 (24,7 - 39,1)	68,4 (60,6 - 75,1)	97,3 (93,4 - 98,9)
Valor de p^ b^	< 0,001	< 0,001	< 0,001
Dominio geográfico			
Lima y Callao	26,3 (24,2 - 33,1)	50,6 (45,3 - 55,9)	85,6 (81,7 - 88,7)
Resto urbano	28,8 (23,8 - 34,4)	51,6 (45,9 - 57,3)	86,0 (80,2 - 90,3)
Rural	25,8 (20,3 - 32,6)	48,9 (41,6 - 56,3)	84,1 (79,8 - 87,6)
Valor de p ^a^	0,623	0,837	0,825
Total	26,8 (22,3 - 29,2)	50,4 (46,7 - 54,0)	85,4 (82,8 - 87,8)

Se incluyó el factor de expansión y las especificaciones muestrales de la encuesta VIANEV 2017-2018.

a Prueba chi cuadrado de Pearson, comparación por sexo y dominio geográfico.

b Prueba chi cuadrado de tendencia lineal, comparación por grupos de edad.

Respecto a la concordancia diagnostica entre el IMC y PA se encontró que fue aceptable; aunque según sexo, los hombres mostraron concordancia moderada (0,49), mientras que las mujeres solo alcanzaron concordancia leve (0,16); por su parte la concordancia diagnóstica entre IMC e ICT en todos los casos resulto leve (Tabla 4).

**Tabla 4:** Concordancia entre diagnóstico de obesidad por índice de masa corporal con perímetro abdominal e índice cintura-talla, según sexo. Perú, 2018.

Indicador	Acuerdo (%)	Kappa de Cohen	Error estándar	Valor de p	Grado de concordancia
Perímetro abdominal					
Sexo					
Mujer	47, 2	0,162	0,023	< 0,001	Leve
Hombre	77,8	0,499	0,040	< 0,001	Moderada
Total	60,8	0,312	0,022	<0,001	Aceptable
Índice cintura-Talla					
Sexo					
Mujer	40,0	0,091	0,017	< 0,001	Leve
Hombre	40,0	0,117	0,021	< 0,001	Leve
Total	40,0	0,111	0,014	<0,001	Leve

La correlación entre las mediciones estandarizadas de IMC y PA resultó pobre; sin embargo, según sexo en varones se obtuvo una correlación moderada, mientras que en mujeres fue pobre (0,98 y 0,87, respectivamente). En el caso de las mediciones estandarizadas de IMC e ICT también se encontró que fue pobre y según sexo se obtuvieron las mismas diferencias (0,90 en hombres y 0,86 en mujeres). En cuanto a la correlación entre valores normalizados de PA e ICT se observó una correlación moderada; los varones mostraron una correlación pobre (0,89), y las mujeres una correlación moderada (0,92). Respecto a los tres pares de comparaciones a nivel general, la correlación entre PA e ICT resultó más alta que las de IMC vs. ICT e IMC vs. PA (Tabla 5).

**Tabla 5:** Medidas de correlación y acuerdo absoluto entre las mediciones estandarizadas de IMC, PA e ICT, según sexo. Perú, 2018.

	Lin	Rho	Valor de p
IMCn y PAn			
Hombres	0,908	0,292	<0,001
Mujeres	0,879	-0,036	0,387
General	0,891	0,081	0,009
IMCn e ICTn			
Hombres	0,900	0,028	0,546
Mujeres	0,863	0,014	0,741
General	0,882	0,063	0,042
PAn e ICTn			
Hombres	0,899	-0,312	<0,001
Mujeres	0,929	0,059	0,153
General	0,916	0,021	0,490

IMCn: valores normalizados del índice masa corporal.

PAn: valores normalizados del perímetro abdominal.

ICTn: valores normalizados del índice cintura-talla.

Lin: coeficiente de correlación de concordancia de Lin para el acuerdo absoluto

Rho: coeficiente de correlación de Spearman entre la diferencia y la media de los valores.

La Figura 1 sobre límites de acuerdo de Bland-Altman muestra que los valores estandarizados de PA e IMC en la población total no tiene relación entre la media de PA e IMC y la diferencia entre ambos indicadores; sin embargo, en los hombres la diferencia aumenta conforme las medias de PA e IMC son mayores, mientras que en mujeres el comportamiento es al revés. En cuanto a los valores estandarizados de ICT e IMC solo se aprecia relación en mujeres; así la diferencia disminuye entre indicadores cuando las medias son más altas. Finalmente, al analizar los valores estandarizados de PA e ICT, en varones se encuentra que los valores de ICT aumentan mientras más grandes sean las mediciones en estos indicadores, mientras que las mujeres muestran un comportamiento ligeramente ascendente.

**Figura 2:** Límites de acuerdo del 95% de Bland-Altman entre los valores normalizados de índice de masa corporal, perímetro abdominal e índice cintura-talla para la población total y según sexo. Perú, 2018.

## DISCUSIÓN

Los resultados del estudio muestran que las correlaciones entre el IMC con PA e ICT fueron pobres, con diferencias entre hombres y mujeres según indicador antropométrico, mientras que la concordancia diagnóstica entre el IMC y el PA fue moderada y del IMC con ICT fue pobre. Respecto a las prevalencias de obesidad fueron significativamente diferentes al aplicar los tres criterios diagnósticos, así según IMC la prevalencia fue de 26,8%, según PA fue de 50,4%, mientras que usando el ICT la prevalencia fue de 85,4%. 

Los valores de concordancia encontrados en el presente estudio son ligeramente menores a lo reportado por Villca *et al.* en Bolivia quienes evaluaron la concordancia entre el IMC, PA e ICT y encontraron un índice de acuerdo de 0,34 entre IMC y PA, mientras que para IMC e ICT el índice fue de 0,28 ^25^. Una revisión sistemática que evaluó las concordancias y correlaciones, aplicando la correlación de Pearson, entre diversos índices antropométricos, concluyó que existe una fuerte correlación entre el IMC y PA, así como IMC e ICT; en tanto que las concordancias fueron moderadas entre estos indicadores antropométricos ^26^. Otros estudios que han analizado la relación entre el IMC y varios indicadores antropométricos e incluso técnicas de imagen y bioimpedancia también encontraron resultados similares. Wilmet *et al*. en Bélgica ^27^ reportaron entre el IMC y PA una correlación de 0,91 para varones y 0,88 en mujeres (p < 0,05), por su parte Chinedu *et al*. en Nigeria encontraron una correlación de 0,63 entre IMC y PA en población adulta ^28^. 

En el presente estudio el IMC mostró una concordancia leve o insignificante con el ICT; sin embargo, este último ha sido reconocido como el índice más preciso para detectar precozmente la hipertensión arterial, diabetes mellitus y síndrome metabólico, así como otras enfermedades cardiovasculares ^29^; esta pobre concordancia del IMC podría reflejar las limitaciones de su uso para la valoración de la adiposidad corporal en población peruana. Se ha cuestionado la aplicabilidad del punto de corte del ICT de 0,5 para diagnosticar obesidad en diferentes edades, sexo y etnias; al respecto la revisión sistemática de Browning *et al.* que incluye 78 estudios en 14 países encontró que el valor de ICT, que abarca todos los riesgos cardiovasculares y metabólicos, fue de 0,50 en ambos sexos ^22^; además, la talla representa la principal variación en los diferentes indicadores antropométricos, la cual es diferente según edad, sexo y etnia. En el caso del ICT, la talla se incluye en la razón, por lo que recogería esta variación y modificaría la interpretación del perímetro abdominal ajustado a la talla del individuo. 

Los resultados del estudio también muestran una correlación moderada entre IMC, PA e ICT y concordancia moderada con PA, lo que indicaría que las estimaciones con estos indicadores antropométricos no son intercambiables, más aún cuando se encuentran diferencias entre hombres y mujeres. Las implicancias de estos resultados abarcan aspectos clínicos como la aplicación de índices antropométricos complementarios al IMC para detectar de manera más precoz la población en riesgo de enfermedades no trasmisibles; asimismo, sugieren la necesidad de revisar y determinar los puntos de corte del IMC más sensibles para predecir enfermedades cardiometabólicas. En general, el IMC es el índice antropométrico que más se usa para determinar la obesidad e indirectamente la cantidad de tejido adiposo, a pesar de que no discrimina el tejido magro de la grasa ^12^. La literatura reciente sobre el diagnóstico de obesidad recomienda el uso de índices de obesidad abdominal pues valoran la ubicación de la grasa e identifican el exceso de grasa central, dando oportunidad al diagnóstico precoz de enfermedades metabólicas ^30^. Por tanto, es necesario evaluar el estado nutricional considerando indicadores de obesidad abdominal para mejorar el diagnóstico, identificar precozmente a la población que requiere tratamiento y evaluar con mayor rigurosidad las intervenciones y políticas públicas contra la obesidad. 

Otros estudios han estimado las prevalencias de obesidad aplicando los diversos índices antropométricos y reportan prevalencias diferentes; así Booth *et al.* en Australia, reportaron mayores prevalencias de obesidad cuando se aplican los criterios de PA e ICT comparados con el IMC ^31^; por su parte Myung *et al*. encontraron prevalencias de obesidad de 3,6%, 26,2% y 43,3% aplicando IMC, PA e ICT, respectivamente ^32^. Asimismo, un estudio en Perú reportó menores prevalencias de obesidad al usar IMC, mientras que aplicando PA e ICT las cifras fueron entre dos a tres veces más ^18^. Estos hallazgos sugieren que la proporción de obesidad aplicando IMC podría estar subestimada en el Perú y que es necesario analizar la especificidad del punto de corte de IMC para la población peruana, así como realizar el diagnóstico de obesidad con indicadores complementarios al IMC. 

El estudio presenta algunas limitaciones, entre ellas que no se incluyó un patrón de oro para establecer un diagnóstico certero de obesidad, se utilizan tres técnicas de diagnóstico, pero existen otras más precisas como la absorciometría fotónica por rayos X (DEXA); además los puntos de corte del PA e ICT aún están en discusión en Perú. Otro aspecto que considerar fue que las mediciones entre los tres criterios diagnósticos dan unidades de medida diferentes, lo que dificulta una comparación directa, por ello se optó por estandarizar las variables de IMC, PA e ICT. 

En conclusión, el estudio encontró que el IMC tiene correlación moderada con el PA e ICT, con diferencias según sexos; asimismo, la concordancia entre el IMC fue moderada con el PA e insignificante con el ICT, que es el mejor predictor de enfermedades cardiovasculares e hipertensión. Los resultados de la correlación y concordancia sugieren que no son indicadores intercambiables y que es necesario evaluar la suficiencia de emplear solo IMC para el diagnóstico de obesidad en nuestro país. Además, las prevalencias de obesidad en el Perú fueron significativamente diferentes al aplicar cada criterio diagnóstico. Se recomienda el uso de índices antropométricos complementarios al IMC que estimen la obesidad abdominal.
